# A community-based knowledge, attitude, and practice survey on rabies among cattle owners in selected areas of Bhutan

**DOI:** 10.1371/journal.pntd.0007305

**Published:** 2019-04-01

**Authors:** Sangay Rinchen, Tenzin Tenzin, David Hall, Frank van der Meer, Basant Sharma, Kinzang Dukpa, Susan Cork

**Affiliations:** 1 Regional Livestock Development Centre, Department of Livestock, Tsimasham, Chukha Bhutan; 2 Department of Ecosystem and Public Health, Faculty of Veterinary Medicine, University of Calgary, Alberta, Canada; 3 National Centre for Animal Health, Department of Livestock, Serbithang, Thimphu, Bhutan; Wistar Institute, UNITED STATES

## Abstract

Rabies remains a disease of significant zoonotic and economic concern in rabies endemic areas of Bhutan. Rabies outbreaks in livestock threaten the livelihoods of subsistence farming communities and pose a potential public health threat. As a part of identifying approaches to prevent rabies in cattle, a Knowledge, Attitude, and Practice (KAP) survey was conducted among cattle owners in selected rural areas of the southern rabies high-risk zone and low-risk zone in eastern Bhutan. Between March and April 2017, 562 cattle owners (281 in the east and 281 in the south) were interviewed using a questionnaire. Eighty-eight percent of the participants had heard of rabies but only 39% of the participants who had heard of rabies had adequate knowledge about rabies. Multivariable logistic regression analysis showed that residing in the south [OR = 9.25 (95% CI: 6.01–14.53)] and having seen a rabies case [OR = 2.46 (95% CI: 1.6–3.82)] were significantly associated with having adequate knowledge about rabies. Based on our scoring criteria, 65% of the total participants who had heard of rabies had a favorable attitude towards rabies control and prevention programs. The participants residing in the east were two times more likely to have a favourable attitude than their counterparts in the south [OR = 2.08 (95% CI: 1.43–3.05)]. More than 70% of the participants reported engaging in farm activities such as examining the oral cavity of sick cattle and assisting cattle during parturition. Only 25% of the participants reported using personal protective equipment while undertaking these activities. Despite a high level of rabies awareness, we observed that there is a lack of comprehensive knowledge about rabies regarding susceptible hosts, transmission routes, the health outcome of rabies infection in humans, and appropriate health-seeking behaviours. This study highlights the need to strengthen rabies education programs in rural communities to address the knowledge gaps that have been identified.

## Author summary

The impacts of rabies outbreaks in cattle and other livestock are multifold. At the local level, economic losses resulting from the direct loss of cattle and the animal’s future productivity is a significant threat to the livelihoods of subsistence farming communities. Besides, rabies in livestock poses a potential public health threat associated with human exposures that result during handling sick animals and dressing carcasses of animals that had died of rabies. Furthermore, the Government incurs costs for responding to outbreaks and providing post-exposure prophylaxis (PEP) to potentially exposed individuals. We conducted a KAP questionnaire surveys among cattle owners in two selected study areas of Bhutan to understand the level of rabies awareness among the cattle owners, their attitude towards rabies prevention and control and to identify existing on-farm practices that can result in potential exposure to rabies in animals and humans. We observed that there was a lack of comprehensive knowledge about rabies especially regarding the post exposure care and outcome of rabies infection in humans and animals. Given that most participants lacked knowledge about clinical signs of rabies in cattle their engagement in practices such as examining oral cavity, assisting cattle during parturition and dressing wounds without wearing minimal protective equipment, was concerning. Our study identifies the need to extend rabies education into the rural communities of Bhutan. Furthermore, our findings reiterate the importance of rabies education as a crucial component of the global initiative to eliminate dog-mediated rabies in humans and animals.

## Introduction

Bhutan is a small Himalayan Kingdom located in South Asia bordered by India in the south, West, and East and the Autonomous region of Tibet in the North. Around 62.2% of its population lives in rural areas and depends on subsistence agriculture and livestock farming [1]. Cattle (*Bos indicus* and *Bos taurus*) are the most commonly reared livestock in Bhutan [2], and they play an important role in sustaining rural livelihoods by serving as a source of food, manure, draught power, and as an immediate source of income. However, infectious diseases remain a threat to livestock production resulting in economic losses due to decreased productivity as well as death of affected animals. Common infectious diseases affecting cattle in Bhutan include foot and mouth disease, rabies, hemorrhagic septicaemia, black quarter, and anthrax. Rabies is one of the most important zoonotic diseases in Bhutan. Despite implementing control measures, the country continues to experience around 17 outbreaks in animals annually, especially in cattle [3]. The Department of Livestock (DoL) under the Ministry of Agriculture and Forests (MoAF) spearheads rabies control program in animals in Bhutan. Apart from conducting mass dog vaccination and animal birth control campaign, rabies awareness education is regularly disseminated to the public via television and radio programs and by observing World Rabies Day annually.

Rabies outbreaks are mainly reported in the southern parts of Bhutan that share a porous border with the neighboring States of India -West Bengal and Assam. This is due to the cross-border movement of dogs [4]. Although the interior part of Bhutan is considered free from dog-mediated rabies, re-emergence of rabies has been observed in the recent past due to incursions of rabid dogs from the bordering towns. For instance, rabies had re-emerged during 2005 and 2016 in the eastern part of the country that share border with an Indian State of Arunachal Pradesh [5, 6]. In Bhutan, rabies is most commonly reported in cattle as a result of spill over infection from dogs [7]. Consequently, several incidents of potential mass human exposure have been reported following handling of rabid animals and carcases during zoo-sanitary measures, and consumption of animal products derived from cattle suspected or confirmed of being infected with rabies [8]. Rabies in cattle results in direct economic losses to the farmers through the loss of cattle and their production, and there is also a cost to the government for responding to outbreaks and providing mass rabies PEP [9]. Although there is a comprehensive Government supported program in Bhutan to control rabies in dogs, no rabies prevention measures are implemented in cattle and other livestock in the rabies endemic areas of Bhutan. To plan appropriate intervention measures to prevent rabies outbreaks in cattle, and to avert subsequent economic losses and public health risks, it is imperative to understand cattle owners’ knowledge and perception about rabies, the prevalent on-farm practices that could predispose exposure to rabies in cattle. Previous community-based studies conducted amongst selected urban residents and dog owners in Bhutan have indicated a high level of awareness and knowledge about rabies [6,10]; however, no studies have been carried out targeting cattle owners and rural households. Therefore, the objectives of this study were to assess and compare the KAP regarding rabies among the cattle owners in selected rural areas of the southern high-risk rabies zone and a rabies low risk zone in the eastern Bhutan. Based on this study findings, a community-based education program will be implemented in the study areas and other identified communities to enhance prevention and control of rabies in humans and animals.

## Materials and methods

### Study areas

**Fig 1:**
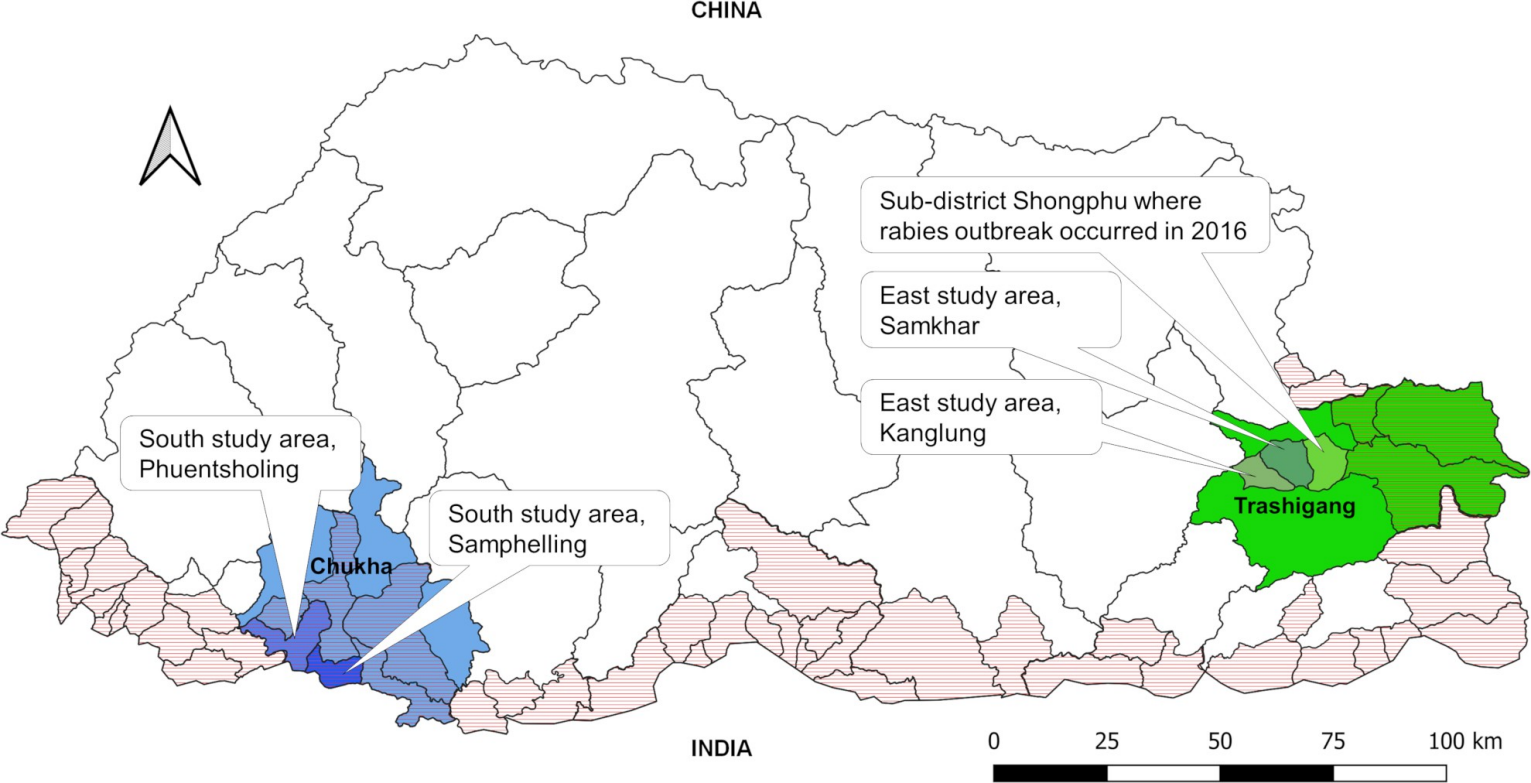
The map of Bhutan showing rabies high-risk areas (the areas with horizontal shade) and the selected study districts and sub-districts. The map also shows a sub-district bordering our study areas in the east that has experienced a major rabies outbreak in October 2016. The map was generated using Quantum GIS software specifically for the purpose of this study [12]. The shapefiles for the political boundary of Bhutan including district and sub-district boundaries were obtained from the National Land Commission of Bhutan.

Administratively, Bhutan is divided into 20 districts (dzongkhags) and 205 sub-districts (gewogs) (Fig 1). Several villages form a sub-district. In this study, two districts—Chukha and Trashigang—were selected based on convenience and purpose [11]. Chukha is a rabies endemic district, while Trashigang is not. Furthermore, as the Regional Livestock Development Centre (RLDC) were in Chukha and Trashigang, it was logistically convenient to recruit and train survey enumerators for the conduct of the study. The sub-districts of Phuentsholing and Sampheling were selected from Chukha district as these sub-districts reports the highest number of rabies outbreaks in animals every year. The sub-district of Kanglung under Trashigang district was included in the study as it falls under a rabies low-risk zone in eastern Bhutan [3]. At the time of our field survey no rabies outbreaks were reported in the southern study areas. However, in October 2016, few months before our study, a major rabies outbreak had occurred in five sub-districts under Trashigang [6]. One of these sub-districts was adjacent to Samkhar, one of our study areas in the east.

### Sample size

The sample size required for comparing two populations was calculated using a web-based sample size calculator designed by the University of British Columbia, Canada (https://www.stat.ubc.ca/~rollin/stats/ssize/b2.html). Since no studies were conducted previously in these selected areas, based on local knowledge, we assumed that 30% and 40% of the cattle owners would have knowledge and awareness about rabies in the eastern and southern areas, respectively. With a confidence level of 95% and the statistical power of 80%, 562 cattle owners (281 from each study area) were required to be selected and interviewed. Twenty-five villages from Phuentsholing and Samphelling, and all the 12 villages from Kanglung sub-district were selected for this study.

The official lists of cattle owners in the selected study areas were collected from the respective District Livestock Offices and were used as the sampling frame. The required number of participants was drawn randomly using Microsoft Excel 2013 (Microsoft excel 2013, Redmond, USA) after assigning a random number to each cattle owner. However, during the field survey, it was observed that some of the selected participants did not own cattle anymore. Some had sold their cattle while others had not replaced their dead cattle. In such cases, the nearest cattle-owning household was selected. For the same reason, in the eastern region, 28 households were interviewed from a neighboring sub-district, Samkhar. In this paper, the southern rabies high-risk study site will be referred to as ‘south’ and the eastern rabies low-risk site as ‘east’.

### Questionnaire survey

A questionnaire comprising five different sections was prepared and used for data collection. Section one comprised questions regarding the participants’ socio-demographic information. Sections two, three, and four comprised questions related to participants’ Knowledge, Attitude, and Practices regarding rabies, respectively. The questions used for collecting information in these four sections were all closed ended. The final section (section five) included both open and close ended questions to determine participants’ farm and farm production characteristics.

Nine livestock personnel working in the Southern and Eastern RLDCs were recruited and trained as survey enumerators. The questionnaire was pretested during mock interviews that were carried out as a part of a survey enumerators’ training program and modified accordingly to improve clarity. The selected households who owned at least one cattle and one of the adult members in households who were 18 years and above, were included for the interview in this study. Prior to start of the interviews, the objectives of the study were explained to the cattle owners. As the survey was targeted in the rural communities with a very low literacy rate, instead of written consent, we sought verbal consent from the selected study participants. The interviews were conducted during March and April 2017 by visiting each selected household in the village.

### Ethics statement

The Conjoint Faculties Research Ethics Board (CFREB), University of Calgary, Canada and the Research Ethics Boards of Health, Ministry of Health, Royal Government of Bhutan approved the study protocol (Approval number REB16-1945 and REBH/Approval/2017/004, respectively).

### Statistical analysis

The questionnaires were checked for completeness before entering the data into Epi Info version 7.2.1.0 [13]. As the data were collected through face-to-face interview by trained survey enumerators, there were only few missing data points and these were identified as “missing data” in the descriptive analysis. The method described by Tack et al., [14] was adapted to categorize participants as either having an ‘adequate knowledge’ or an ‘inadequate knowledge’ about rabies. Participants were scored on five questions to assess their knowledge about rabies (S1 Table). It was assumed that the participants with adequate knowledge about rabies would be aware of notable clinical signs in a rabid dog, appropriate health-seeking behaviour upon exposure, the health outcome of contracting rabies in humans, a potential rabies reservoir, and at least one mode of rabies transmission. For identifying a rabid dog, irrespective of what other signs participants reported, if any of the five clinical signs listed in the questionnaire (salivation, aggressiveness, biting, aimless movement, and paralysis and death) was mentioned, the participants were considered being able to identify a rabid dog. Since identifying a rabid dog, seeking proper health care after exposure, and knowing the health outcome in humans suffering from rabies is crucial in preventing human deaths due to rabies, participants had to answer these questions correctly. If any of these questions were answered incorrectly, or if participants were not sure of the answer, irrespective of the total score obtained, they were categorized as not having adequate knowledge about rabies. Therefore, a participant with an adequate knowledge would score a minimum of five points, three from the questions on rabies signs in dogs, disease outcome and response upon exposure and two from the questions regarding rabies reservoir and modes of rabies transmission.

The method of assessing participants’ attitude was adapted from Dhimal et al., [15]. Seven questions were used to assess participants’ attitude towards rabies prevention in humans and animals (S2 Table). Participants could choose an answer on a qualitative scale of very low to very high for each question. If the response was “High” and “Very High” participants were awarded one point while for the rest of the responses “Very low”, “Low” and “Medium” no points were awarded. From seven questions, a participant could secure maximum of seven points. Participants were then categorized as having a favourable attitude or an unfavorable attitude based on 80% of the total score as the cut-off, as described by Dhimal et al., [15].

The data entered into Epi Info database were screened and prepared for analysis in Microsoft Excel 2013 (Microsoft excel 2013, Redmond, USA). Analyses were conducted using R packages “dplyr”, “descr”, “forcats” “lmtest”, “LogisticDx”, “ggplot2” and “stringr” within R statistical software [16–23]. For analysis, the variable age was categorized as 18–35, 36–53 and >53 years and the variable education as ‘educated’ or ‘uneducated. Descriptive analysis was conducted for the entire dataset. The frequencies of the categorical socio-demographic variables between the two study areas were compared using Pearson’s chi-square test. The mean age of the participants in the two study areas was compared using student’s t-test.

To assess the association between the socio-demographic variables and the binary outcome variables, 1) participants’ knowledge about rabies (adequate vs inadequate) and 2) participants’ attitude towards rabies control and prevention programs (favourable vs unfavourable), a series of univariable logistic regression analysis were carried out. Variables with p-value (≤ 0.2) were selected for multivariable logistic regression analysis. A forward stepwise method of multivariable logistic regression analysis was performed by adding foremost the variable that had the smallest p-value in the univariable analyses. Subsequently, each of the remaining variable was added. The variables with p≤0.05 were retained in the final model. The fit of the final multivariable logistic regression model was assessed by conducting Hosmer and Lemeshow test [11]. The odds ratio (OR) and its 95% confidence interval (CI) of the variables associated with the outcome variables were derived from the final multivariable logistic regression model. Confounding was assessed by adding the variables that were not included in the final model as described by Lindahl et al. (24). If the coefficients of the significant variables changed by more than 25%, the added variable was considered to be a confounder. No confounders were observed in our analyses.

## Results

### Socio-demographic characteristics

Five hundred and sixty-two participants, 281 each from the south and the east, were interviewed. The response rate was 100%. There was no significant difference (p = 0.17) between the mean age of participants in the south (45.8 years) and in the east (47.5 years). The Chi-square analysis showed that the number of female participants (p<0.001), participants having children going to school (p<0.01), participants owning only exotic breeds of cattle (p<0.001), and owning less than 4 cattle (p<0.001) were significantly higher in the eastern study areas. The detail of the socio-demographic characteristics in the two study areas is presented in Table 1.

**Table 1:** Socio-demographic characteristics of the participants in the selected southern and eastern areas of Bhutan.

Variables	Categories	South (n = 281)	East (n = 281)	Total (n = 562)	χ^2^ p-value
**Gender**	Male	205 (73)	99 (35)	304 (54)	<0.001
	Female	76 (27)	182 (65)	258 (46)	
**Age**	18–35	76 (27)	55 (20)	131 (23)	0.070
	36–53	114 (41)	136 (48)	250 (45)	
	Above 53	91 (32)	90 (32)	181 (32)	
**Education level**^϶^	No education	176 (62.8)	173 (61.3)	349 (62)	0.794
	Non-Formal Education	9 (3.2)	48 (17.4)	57 (10.3)	
	Primary level	52 (18.5)	29 (10.3)	81 (14.4)	
	Lower Secondary	24 (8.5)	8 (2.8)	32 (5.7)	
	Higher Secondary	17 (6)	6 (2.1)	23 (4.1)	
	University graduate	2 (0.7)	1 (0.4)	3 (0.5)	
	Post-graduate	1 (0.4)	0 (0)	1 (0.2)	
	Buddhist studies	0 (0)	16(5.7)	16 (2.8)	
**Occupation**	Farmer^¶^	237 (84)	258 (92)	495 (88)	0.319
	Housewife^¶^	21 (8)	7 (3)	28 (5)	
	Government employee	4 (1)	2 (1)	6 (1)	
	Businessman	7 (3)	5 (2)	12 (2)	
	Private/corporate sector	8 (3)	3 (3)	11 (2)	
	Others	4 (1)	6 (2)	10 (2)	
**Type of settlement**	Rural	251 (89)	281 (100)	532 (95)	<0.001^§^
	Urban	30 (11)	0 (0)	30 (5)	
**Cattle type owned**	Only exotic breeds^¥^	133 (47)	231 (82)	364 (65)	<0.001
	Both the breeds ^ж^	148 (53)	50 (17)	198 (35)	
**Cattle holding per household**	Below 4	135 (48)	177 (63)	312 (55)	<0.001
	Above 4	146 (52)	104 (37)	250 (45)	
**School going children**	No	93 (33)	62 (22)	155 (27)	<0.01
	Yes	188 (67)	219 (78)	407 (73)	
**Owning dog**	No	152 (46)	151 (46)	303 (54)	0.9332
	Yes	129 (54)	130 (54)	259 (46)	

The figures in the brackets represent percentages

^϶^ The participants falling under “No education” were considered “Un-educated” while the rest were considered “Educated” for the purpose of statistical analysis.

^¶^ For analysis purpose, “Farmer” and “Housewife” were considered as “Unemployed” and the rest as “Employed”.

^§^ p-value of a Fisher’s exact test

^¥^ Exotic cattle breed includes pure and cross-bred Jersey, Holstein Friesian, and Brown Swiss

^Ж^ Both the exotic breeds mentioned above and the indigenous cattle

### Knowledge about rabies

**Fig 2:**
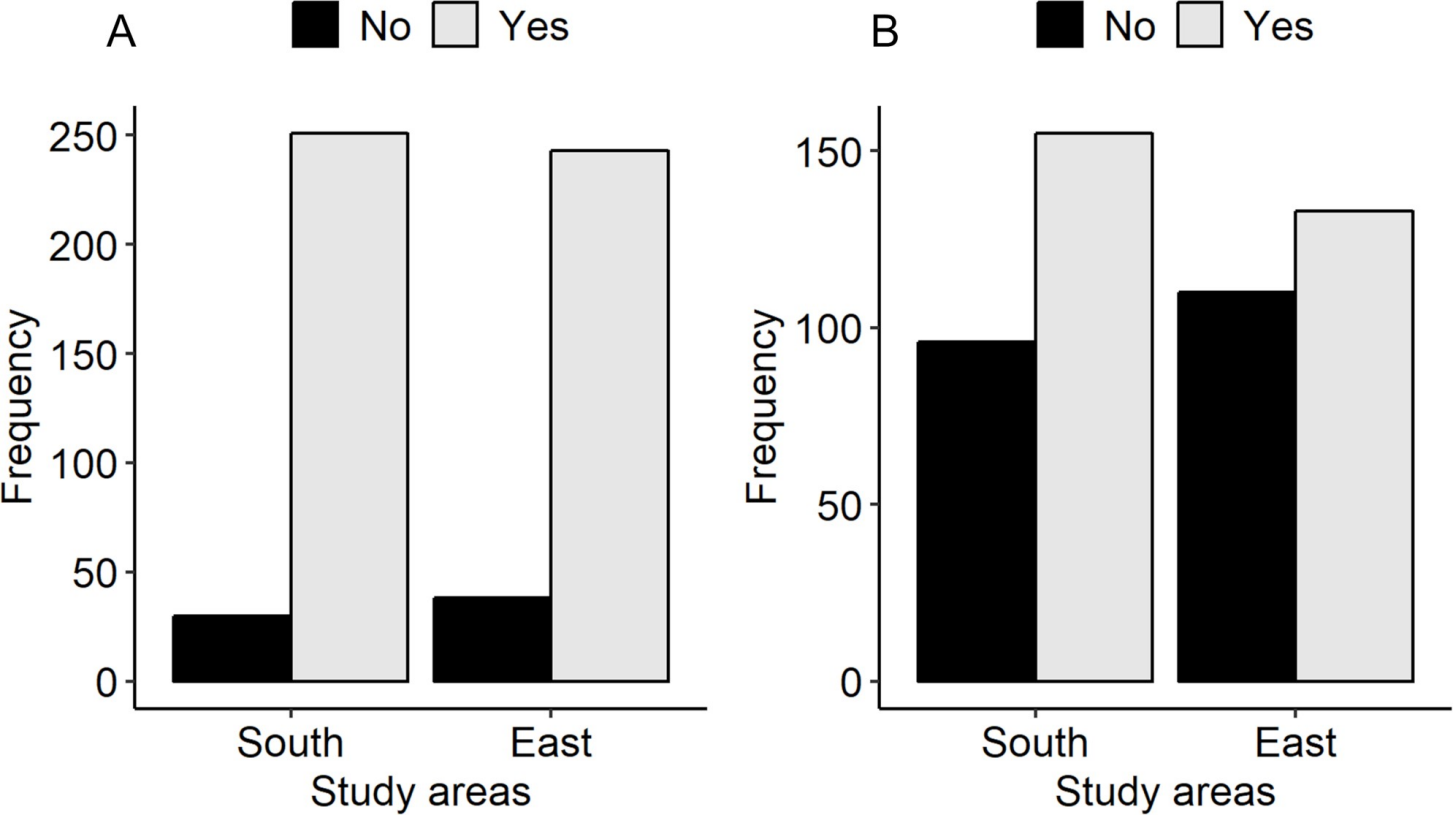
Participants who had heard of rabies (A) and seen a rabies case in animals or humans (B) categorized by study areas (n = 562).

Overall, 494 of the 562 (88%) participants had heard of rabies (Fig 2A). In the south, 251 (89%) participants had heard of rabies whereas, in the east, 243 (86%) participants had heard of rabies. Having heard about rabies was not associated with the place of residence. Among the participants who had heard of rabies, 106 (22%) participants did not know the signs shown by a rabid dog while 223 (45%) reported that they would not be able to identify rabies in cattle. While the participants in the east were less likely to know the clinical signs of rabies in dogs (p<0.001), there was no significant difference in the percentage of participants between the two study areas who could not identify rabid cattle (107 (43%) in the south and 116 (48%) in the east). A total of 288 (58%) participants who had heard of rabies had seen a rabies case, either in a dog, a human or in livestock. The place of residence was not associated with seeing a rabies case. In the south, 155 (62%) participants had seen a rabies case while 133 (55%) participants in the east had seen a rabies case (Fig 2B).

The number of participants who knew about preventive rabies vaccination in dogs was 446 (90.3%). The observation was not statistically associated with the place of residence (224 (89%) in the south and 222 (91%) in the east). In total, 370 (75%, n = 492, two missing) participants were not aware of pre-exposure prophylactic vaccination (PrEP) in cattle. One-hundred and fifty-seven (63%, n = 250, one missing) participants in the south and 213 (88%, n = 240, one missing) participants in the east reported not knowing about PrEP in cattle. Furthermore, 334 (68%, n = 493) participants reported not knowing about the PrEP option for humans.

In total 441 (89%) participants reported that rabies can be effectively prevented in humans after a potential exposure. However, only 399 (81%) participants knew about the standard PEP regimen which consists of washing the bite wound with soap and water followed by injection of four to five doses of rabies vaccine. Of the remainder, washing the bite wound with soap and water as standard PEP regimen was reported by 16 (3%) participants, washing the bite wound and a shot of vaccine by 32 (7%), and 47 (9%) reported not knowing about the regimen. The proportion of individuals aware of the standard PEP regimen was higher in the southern study group than the eastern study group (p<0.0001).

**Fig 3:**
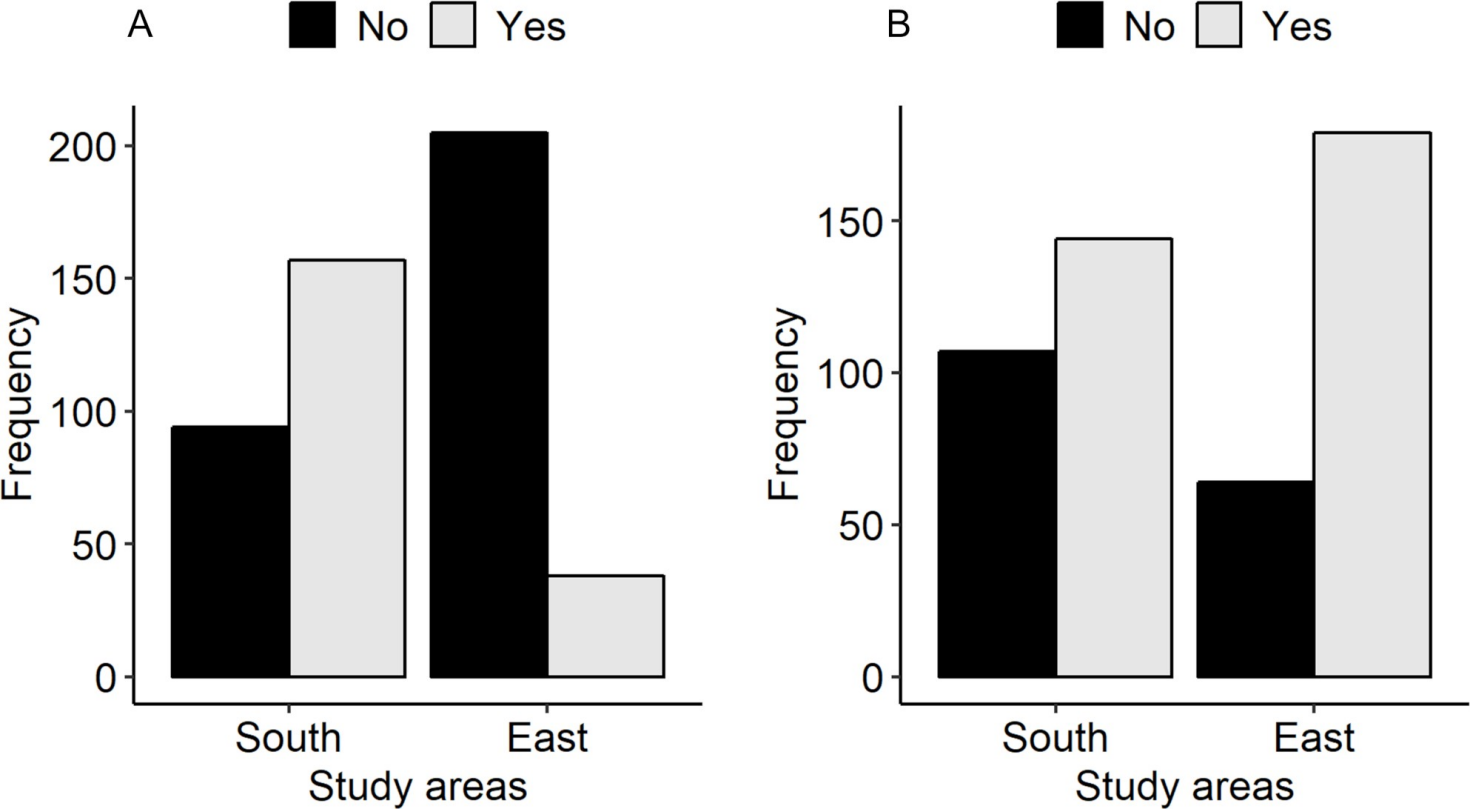
Participants who had “adequate vs inadequate knowledge about rabies” (A) and a “favourable vs unfavourable attitude towards rabies control and prevention programs” (B) categorized by study areas (n = 494).

Among the participants who had heard of rabies, 195 (39%) participants had adequate knowledge about rabies (Fig 3A). The multivariable regression analysis demonstrated that individuals residing in the south were 9.25 times more likely to have adequate knowledge about rabies than their counterparts in the east [OR = 9.25 (95% CI: 6.01–14.53)], while the individuals who had seen a rabies case were 2.5 times more likely to have adequate knowledge about rabies than those who had not seen a rabies case [OR = 2.46 (95% CI: 1.6–3.82)] (Table 2).

**Table 2:** Logistic regression analysis to understand the association between the explanatory variables and the binary outcome variable–“having adequate knowledge about rabies or not”.

Variables	Categories	Adequate knowledge		Total	OR (95% CI)	Adjusted OR (95% CI)
		No	Yes			
**Education**	Educated	106	81	187	reference	
	Un-educated	193	114	307	0.77 (0.53–1.12)	
**Gender**	Female	162	54	216	reference	
	Male	137	141	278	3.08 (2.11–4.57)	
**Place of residence**	East	205	38	243	reference	Reference
	South	94	157	251	9.01 (5.91–14.0)	9.25 (6.01–14.53)
**Seen rabies**	No	148	58	206	reference	Reference
	Yes	151	137	288	2.32 (1.58–3.41)	2.46 (1.6–3.82)

OR = Odds Ratio

CI = Confidence interval

### Attitude towards rabies prevention

Based on our scoring criteria, 323 (65%) of the total study participants who had heard of rabies had a favorable attitude towards rabies control and prevention programs (Fig 3B). In the south, 144 (57%) participants had a favorable attitude while 179 (73.7%) in the east. The multivariable logistic regression analysis showed that an individual residing in the east was 2.08 times more likely to have a favorable attitude than a person residing in the south [OR = 2.08 (95% CI: 1.43–3.05)] (Table 3).

**Table 3:** Logistic regression analysis to understand the association between the explanatory variables and the binary outcome variable–“having favourable attitude towards rabies control and prevention program or not”.

Variables	Categories	Favourable attitude		Total	OR (95% CI)	Adjusted OR (95% CI)
		No	Yes			
**Age**	>53	51	110	161	Reference	
	18–35	47	62	109	0.61 (0.37–1.01)	
	36–53	73	151	224	0.96 (0.62–1.48)	
**Place of residence**	South	79	127	206	Reference	reference
	East	92	196	288	2.08 (1.43–3.05)	2.08 (1.43–3.05)
**Seen rabies**	No	107	144	251	Reference	
	Yes	64	179	243	1.33 (0.91–1.93)	

OR = Odds Ratio

CI = Confidence interval

### Self-reported farm practices

Only 81 (14%) participants reported stall feeding their cattle (South 79 (28%); east 2 (1%)) while 155 (28%) reported practicing extensive grazing, 174 (31%) semi-extensive grazing and 148 (26%) tethering cattle. Of the 555 participants (7 missing), 143 (26%) reported not having a shed for their cattle while 240 (43%) reported having a temporary shed. Only 172 (31%) participants reported having reliable cattle shed with concrete flooring and appropriate roofing. The number of participants having reliable cattle shed was more in the south compared to the east (108 (39%) and 64 (23%), respectively.

Five-hundred and seventeen participants (92%) stated that they would either report a cow that had died of illness to a livestock official or they would bury the carcass. Of these, 427 (76%) participants reported that they would bury the carcass while 90 (16%) would report to a livestock official. However, 15 (3%) participants reported dressing the carcass to sell the meat, 15 (3%) reported dressing carcass for family consumption, and 15 (3%) reported selling the whole carcass.

In total, 490 (87%) participants reported assisting their cattle during parturition, 512 (91%) reported handling sick cattle, 404 (72%) reported examining oral cavity, 471 (84%) reported dressing wounds in animals, and 138 (25%) reported dressing carcasses. The proportion of participants who dressed cattle carcasses was twice as much in the east than the south (33 and 16% respectively). Among the participants who reported engaging in the mentioned activities, 25 (18%) reported using minimal personal protective equipment (PPE) while dressing carcass (15 in the south and 10 in the east). Similarly, 121 (26%) reported using PPE while dressing a wound in their cattle, 114 (23%) while assisting parturition, 88 (22%) while examining oral cavity, and 127 (25%) while handling sick animals. However, in both the study areas, more than 95% of the participants reported washing their hands after engaging in on-farm activities.

### Source of rabies information

**Fig 4:**
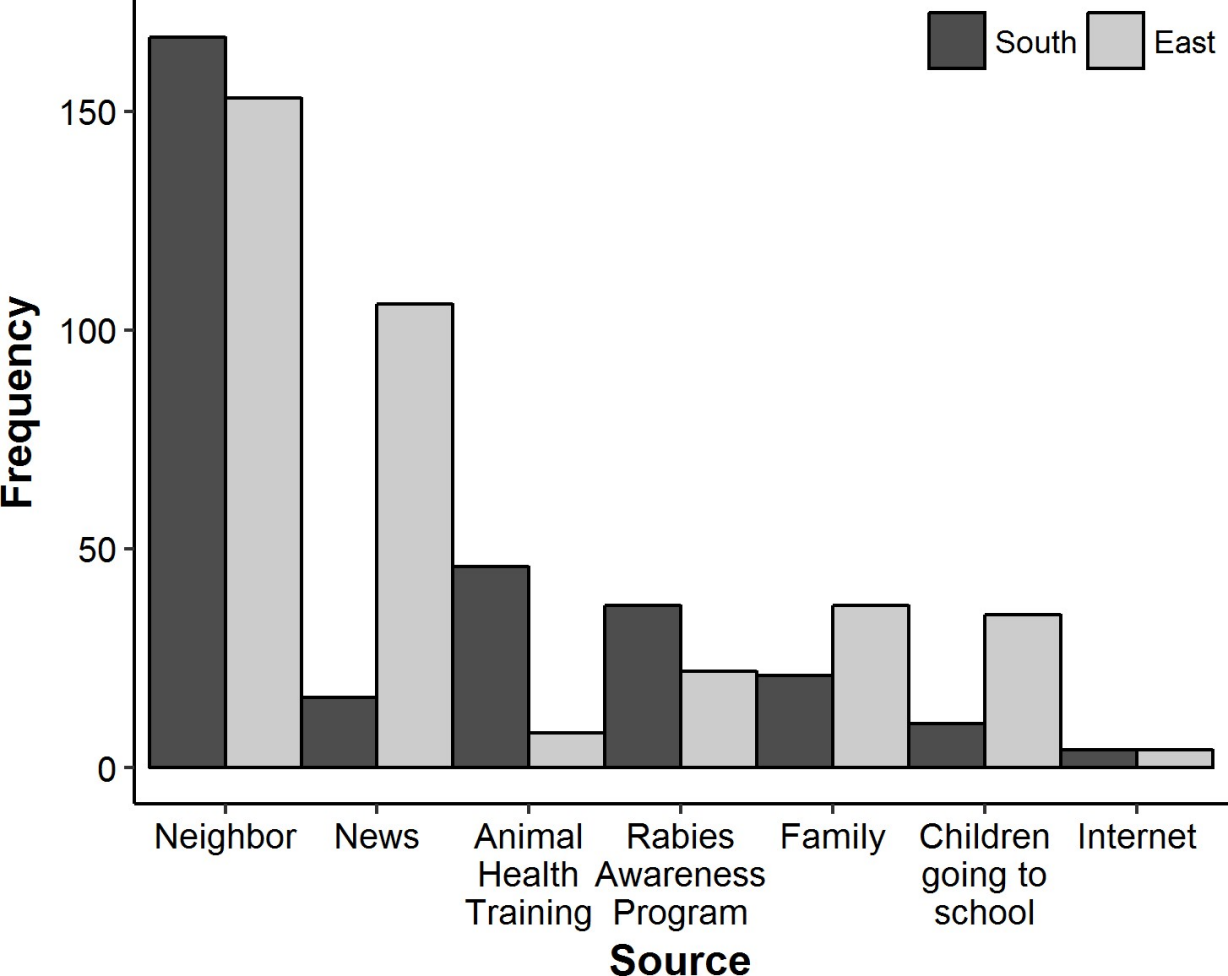
Common sources of rabies information reported by the study participants (Note: Since the participants had heard of rabies from multiple sources, the total frequency of this graph will sum up to more than 494 -the total number of participants who had heard about rabies).

Three-hundred and fifteen participants (64%) reported the neighbor as the most common source of rabies information followed by formal news media such as television, radio, and newspaper (122 (23%)). One hundred and thirteen (22%) participants reported hearing about rabies from animal health training programs and rabies awareness campaigns, 58 (12%) from family members, 45 (10%) from school going children, and 8 (2%) through internet (Fig 4)

## Discussion

To our knowledge, this is the first KAP survey targeting cattle owners in rural communities of Bhutan regarding rabies. The level of awareness about rabies was uniformly high in both the southern and eastern study areas which is in agreement with the findings reported from community-based surveys conducted in the urban areas of eastern (Rangjung) and southern (Gelephu) towns of Bhutan [6,10]. The level of awareness about rabies among participants in both the study areas reflects the continued efforts made by the Government of Bhutan in imparting regular rabies awareness education. However, we identified some knowledge gaps amongst the participants in both the rural study areas. For instance, some of the participants reported not knowing the correct practice to be adopted after a potential exposure. Even among the participants who would seek medical help after a potential exposure, a substantial proportion did not know that a correct PEP regimen involved wound washing followed by four to five doses of rabies vaccine injection spread over several weeks. The observation that participants were unaware of the PEP regimen could be part of the reason that potentially exposed individuals do not complete the required course. In a recent study conducted to understand the use and distribution of human rabies PEP vaccine in Bhutan, it was estimated that about 40% of exposed individuals did not complete the recommended PEP regimen [25].

As expected, it was observed that a large proportion of study participants did not know about PrEP option for humans. This could be because current rabies education programs, at the community level, are focused on promoting mass dog vaccination and appropriate health-seeking behavior after a potential exposure. Furthermore, the WHO recommends mass PrEP only in areas where rabies is very common and there is an extremely high incidence of dog bites or in high risk groups such as veterinarians and wild animal handlers [26].

In Bhutan, most of the livestock vaccinations are provided free of cost by the government. However, as there is no government policy supporting PrEP in cattle and other livestock species, they are not routinely vaccinated against rabies. The current rabies control programs in Bhutan are focused on eliminating dog-mediated rabies through mass dog vaccination. This may explain our finding of a high proportion of study participants being unaware of rabies PrEP in cattle. To mitigate the economic and public health impact of rabies, the World Organization for Animal Health (OIE) recommends vaccinating cattle and other livestock against rabies in rabies-endemic areas where the risk of exposure to rabies is high [27]. It is known that mass dog vaccination is a cost-effective and proven tool for the elimination of rabies virus in dogs and the Government of Bhutan expects to continue to vaccinate the dogs as guided by the current policy [3]. However, in areas of the country where the risk of exposure to rabies remains high, for example, as a result of cross-border incursions, PrEP could be explored as an option to prevent rabies infection in cattle and other livestock.

A lower than expected proportion of study participants had adequate knowledge about rabies according to our criteria. A lack of comprehensive knowledge about rabies has been reported from a similar KAP survey in Tanzania [28]. The multivariable logistic regression analysis showed that the people residing in the south and having seen rabies was positively associated with having adequate knowledge about rabies. The observation of a higher proportion of participants in the south having adequate knowledge about rabies reflects the extra emphasis that the government of Bhutan has placed on targeting awareness education and control program in higher risk areas due to the high prevalence of rabies. In our study, we selected one of the sub-districts adjoining a sub-district in eastern Bhutan that had recently experienced rabies outbreak. Finding a low proportion of participants in this community having adequate knowledge about rabies in the east highlights the need to extend rabies education programs into the low-risk areas adjoining potentially high-risk zones. Enhancing veterinary surveillance in these areas and educating the public about rabies can result in a better level of reporting and higher vaccination coverage in dogs.

Active community engagement is crucial in effective implementation of rabies control programs in communities [29]. As the study area in the east is currently considered free of rabies, the observation that participants have a favorable attitude to rabies prevention and control program could be associated with the fear of rabies incursion and its associated consequences in the areas where rabies did not exist. However, we acknowledge that this finding could have been biased by the recent government intervention in creating extensive awareness among public in the eastern high-risk areas of Bhutan following a major rabies outbreak that lasted for almost a year [6]. Meanwhile, given the high prevalence of rabies in the south, the finding of a lower proportion of participants having favorable attitude is surprising. Although regular awareness programs are conducted in the southern rabies endemic areas, these interventions are largely targeted to the urban residents and schools. Our finding highlights the importance of extending rabies education in rural communities adjoining high and medium-risk areas.

Besides vaccination, reducing contact between cattle and free-roaming dogs through improving cattle management—reducing free grazing and providing housing—can prevent potential rabies exposures. However, we observed that a low proportion of participants reported having a proper cattle shed while a large proportion reported letting their animals roam free with little or no monitoring. Considering the entrenched age-old practice of extensive or semi-extensive cattle rearing and the socio-economic background of the marginal cattle owners, bringing about an immediate improvement in cattle management practices would not be feasible. Nevertheless, heightened monitoring of cattle brought about by enhanced communications between the livestock officials and cattle farmers during the local outbreaks can be crucial in preventing exposures and subsequent rabies infection in cattle.

Annually, the public health sector of Bhutan spends around six million Ngultrum (1 USD = Nu. 68) for providing free rabies PEP to potentially exposed individuals. Between 2009 and 2012, 18,813 individuals were provided PEP, of which 868 received PEP for consuming animal products such milk and meat derived from rabid or suspected rabid cattle due to lack of awareness on rabies [30]. Mass potential exposure of humans due to rabies in cattle resulting from consuming raw milk and handling sick cattle has also been reported from other parts of the world [31,32]. Pasteurization of milk can eliminate the risk of rabies and also safeguard consumers from other milk-borne pathogens such as bovine tuberculosis and brucellosis [33]. However, due to the taste and convenience, raw milk is still preferred over boiled milk in some parts of the world [34]. The risk of contracting rabies upon consuming raw milk from a rabid animal remains theoretically possible [31]. Since the uncertainty around the risk of rabies transmission through consumption of milk is high, in most cases all exposed individuals are provided PEP. However, in this study, only 2% of the participants reported consuming raw milk while the rest reported not consuming or either boiling or fermenting before consumption. This contrasted with the observations from Tajikistan and Zimbabwe where 30% and 41.5% participants respectively, reported consuming raw milk [24,35].

A very small proportion (8%) of the participants reported that they would either dress a carcass (for sale or consumption) or sell the whole carcass of a cattle that had died of a disease. Contrary to the report from a survey of cattle owners in Senegal, where 89.6% of participants paid no attention to proper disposal of animal carcasses [36], 92% of the participants in our study reported desirable cattle carcass disposal practices such as burying the carcass and reporting to a livestock official.

Engagement in ‘on-farm’ practices such as examining oral cavities, dressing a wound, handling sick animals, and assisting cattle during parturition without wearing minimal personal safety equipment such as gloves and mask was commonly reported. Similar reports of assisting animals during parturition without wearing protective gloves were reported from India and Senegal [36,37]. Since rabies can also be transmitted from broken skin and mucous membranes contacting infectious body fluids, particularly saliva, engaging in these activities can also lead to potential exposure to other zoonotic diseases including leptospirosis and brucellosis. Brito et al., [38] reported a case of a veterinarian in Brazil contracting rabies while handling rabid herbivores (bovine and caprine). Similarly, a case of an animal health worker contracting rabies after inserting abraded hands without protection into a mouth of a rabid cow was reported from Iran [39]. In the same report, Simani et al., [39] report mortality due to rabies in three shepherds, a father, and his two sons, after handling bite wounds in their animals inflicted by a rabid wolf. Further, there is also a report of butcher contracting rabies after skinning a calf that died of unspecified neurological signs [40]. However, washing hands with soap and water after engaging in these activities can minimize the risk of contracting rabies and other zoonotic diseases. In this survey, 95% percent of the participants who reported engaging in the aforementioned activities indicated that they washed their hands after handling animals.

As reported elsewhere, our study participants reported neighbors as the most common source of rabies information [28,41]. This could be due to frequent interactions in rural communities facilitated by close social settings where helping each other for farm and agricultural works and inviting neighbors over social activities is still a very common practice in Bhutan. Therefore, disseminating health messages through identified influential community members could be instrumental in ensuring that the key public health and disease prevention messages are distributed effectively across communities. One disadvantage of this means of disseminating health information, however, is the likelihood of information being distorted as it is passed on from one person to the other.

Like any other observational studies, our study has some limitations. During our study, a few sub-districts adjoining the study areas in the east had experienced rabies outbreaks—October 2016 through December 2017, and extensive awareness education programs were conducted as a part of the response measures. This intervention could have biased the information, especially regarding the awareness about rabies. Although the households were randomly selected, during the survey some of the households, which no longer owned cattle had to be substituted by the next cattle-owning household. Furthermore, the survey enumerators interviewed those they met at home during their visit. As the interview in the east coincided with potato plantation, a major cash crop grown in these areas, most of the men from the households were out in the field. Therefore, there were more female participants as opposed to the higher proportion of male participants in the south. The non-uniform gender representation in two study areas could have biased our findings. In addition, response bias would have resulted due to the use of multiple choice answers (close end questions) to collect information regarding the participants’ knowledge about rabies and their attitude towards rabies control and prevention programs. We also acknowledge that our findings regarding the “on-farm” practices could be biased as these were self-reported practices. Finally, due to convenience and purposive sampling at district, sub-district and village level, the findings have weak external validity. Given these limitations, some caution needs to be placed in generalizing our findings beyond the study areas. Nevertheless, this study provides an overview of the Knowledge, Attitude, and Practices of cattle owners regarding rabies in two regions of rural Bhutan.

### Conclusion

This study has shown that there is a lack of comprehensive knowledge about rabies, especially regarding the modes of transmissions, identifying rabid animals (dogs and cattle), the severity of the outcome of rabies infection in humans, and the recommended PEP regimen, in both the study areas. Therefore, future rabies education programs should focus on educating communities on the severity of the outcome of rabies infection in humans and animals, modes of transmission, appropriate health-seeking behaviours after a potential exposure, and good farm practices. It is also essential to include topics such as the importance of responsible animal (dogs and livestock) ownership and community participation in rabies control programs. The observation that participants in the eastern rabies low-risk areas have less knowledge about rabies compared to the participants in the areas within the rabies high-risk zone underlines the need to extend rabies education programs to the communities close to the borders and other areas where rabies incursions are more likely.

## Supporting information

S1 Checklist Strobe checklist used for our study. (DOC)

S1 Table Questions used for assessing particpant’s knowledge about rabies. (DOCX)

S2 Table Questions used for assessing participant’s attitude towards rabies prevention and control in humans and animals. (DOCX)

S1 Dataset Data collected during the survey and used for analysis. (XLSX)
