# Supplementary material for: A community-based knowledge, attitude, and practice survey on rabies among cattle owners in selected areas of Bhutan
Source: PLoS Negl Trop Dis. 2019 Apr 1;13(4):e0007305. doi: 10.1371/journal.pntd.0007305 (PMC6459539; doi:10.1371/journal.pntd.0007305)
Supplement: S1 Table — (DOCX) [file pntd.0007305.s002.docx]

**S1. Table. Questions used for assessing participants’ knowledge about rabies**

| **Questions** | **Score** | **Criteria** |
| --- | --- | --- |
| Do you know the signs and symptoms of rabies in dogs? *  (*salivation, aggressiveness, biting, aimless movement, and paralysis and death*) | 1 | A point was awarded if at-least one correct clinical sign was mentioned, otherwise no point awarded |
| What should be done if bitten by any of these animals (Dog, cat, cattle, horse)? * | 1 | A point was awarded if participants reported they would wash the bite wound with soap and water and visit health center, otherwise no point awarded |
| What will be the outcome in humans infected with rabies? * | 1 | A point was awarded if participants reported “death” as an outcome of rabies, otherwise no point awarded |
| Potential rabies reservoir | 1 | A point was awarded if participants mentioned at-least one potential reservoir, otherwise no point awarded |
| Transmission route | 1 | A point was awarded if participants mentioned at-least one correct route of rabies transmission, otherwise no point awarded |

*If participants answered any of these questions incorrectly, irrespective of the score they obtained, they were categorized as not having adequate knowledge about rabies
