# Supplementary material for: A community-based knowledge, attitude, and practice survey on rabies among cattle owners in selected areas of Bhutan
Source: PLoS Negl Trop Dis. 2019 Apr 1;13(4):e0007305. doi: 10.1371/journal.pntd.0007305 (PMC6459539; doi:10.1371/journal.pntd.0007305)
Supplement: S2 Table — (DOCX) [file pntd.0007305.s003.docx]

**S2. Table. Questions used for assessing participants’ attitude towards rabies prevention and control in humans and animals**

| **Questions** | **Score** | **Criteria** |
| --- | --- | --- |
| **Benefit** of vaccinating dogs in preventing rabies outbreaks | 1 | A point was awarded if the response was “High” and “Very high”, otherwise no point awarded |
| **Benefit** of helping Livestock sector during rabies vaccination and dog sterilization programs | 1 | A point was awarded if the response was “High” and “Very high”, otherwise no point awarded |
| **Benefit** of proper housing of cattle in preventing rabies in cattle | 1 | A point was awarded if the response was “High” and “Very high”, otherwise no point awarded |
| **Benefit** of wearing protective cloths while handling sick animals, dressing carcass and carrying our other farm activities in preventing you from exposure to rabies | 1 | A point was awarded if the response was “High” and “Very high”, otherwise no point awarded |
| **Convenience/ease** to help Livestock sector during dog vaccination and sterilization programs | 1 | A point was awarded if the response was “High” and “Very high”, otherwise no point awarded |
| **Convenience/ease** to follow good farm practices (wearing protective cloths, washing hands) | 1 | A point was awarded if the response was “High” and “Very high”, otherwise no point awarded |
| **Convenience/ease** to properly house your cattle during rabies outbreak period | 1 | A point was awarded if the response was “High” and “Very high”, otherwise no point awarded |
